# Mapping quantitative trait loci for T lymphocyte subpopulations in peripheral blood in swine

**DOI:** 10.1186/1471-2156-12-79

**Published:** 2011-09-16

**Authors:** Xin Lu, Jian-Feng Liu, Yuan-Fang Gong, Zhi-Peng Wang, Yang Liu, Qin Zhang

**Affiliations:** 1 Key Laboratory of Animal Genetics Breeding and Reproduction, Ministry of Agriculture, College of Animal Science and Technology, China Agricultural University, Beijing 100193, China; 2 State Key Laboratory for Infectious Disease Prevention and Control, National Institute for Communicable Disease Control and Prevention, Chinese Center for Disease Control and Prevention, P.O. Box 5, Changping, Beijing 102206, China; 3 Department of Animal Science, Hebei Normal University of Science and Technology, Changli, Hebei 066600, China

**Keywords:** T lymphocyte subpopulations, quantitative trait loci, swine

## Abstract

**Background:**

Increased disease resistance through improved general immune capacity would be beneficial for the welfare and productivity of farm animals. T lymphocyte subpopulations in peripheral blood play an important role in immune capacity and disease resistance in animals. However, very little research to date has focused on quantitative trait loci (QTL) for T lymphocyte subpopulations in peripheral blood in swine.

**Results:**

In the study, experimental animals consist of 446 piglets from three different breed populations. To identify QTL for T lymphocyte subpopulations in peripheral blood in swine, the proportions of CD4+, CD8+, CD4+CD8+, CD4+CD8-, CD4-CD8+, and CD4-CD8- T cells and the ratio of CD4+:CD8+ T cells were measured for all individuals before and after challenge with modified live CSF (classical swine fever) vaccine. Based on the combined data of individuals from three breed populations, genome-wide scanning of QTL for these traits was performed based on a variance component model, and the genome wide significance level for declaring QTL was determined via permutation tests as well as FDR (false discovery rate) correction. A total of 27 QTL (two for CD4+CD8+, one for CD4+CD8-, three for CD4-CD8+, two for CD4-CD8-, nine for CD4+, two for CD8+, and eight for CD4+:CD8+ ratio) were identified with significance level of *FDR *< 0.10, of which 11 were significant at the level of *FDR *< 0.05, including the five significant at *FDR *< 0.01.

**Conclusions:**

Within these QTL regions, a number of known genes having potential relationships with the studied traits may serve as candidate genes for these traits. Our findings herein are helpful for identification of the causal genes underlying these immune-related trait and selection for immune capacity of individuals in swine breeding in the future.

## Background

Infectious diseases cause many serious economic and welfare problems in current swine industry and some of them belong to zoonoses, leading to potential risks to human health. Currently the main way of dealing with infectious diseases in swine is to prevent infection through hygienic measures, vaccination and supplementation of antibiotic in feed. However, these measures could not solve this problem completely [1,2]. Alternatively, breeding for enhanced immune capacity and improved resistance to infectious diseases provides a promising way to address potential disease issues from a genetic perspective. Moreover, swine are increasingly used as a large animal model for several human diseases [3-6]. Therefore, the functions of porcine immune system become more and more interesting both in basic and applied research.

The immune system plays an essential role in disease resistance of animals. Lymphocytes, also called white blood cells, have been wildly recognized as a major component of the adaptive immune system, assuming very crucial responsibility for immunity as well as allergy. Lymphocytes are basically divided into two categories, namely T and B lymphocytes, each responsible for a particular branch of the immune system. T-lymphocytes are mostly responsible for fighting microbes, antigens or foreign substances inside the cells, triggering so-called cell-mediated immunity.

CD4+ and CD8+ T cells are two important subsets of T lymphocytes, which are highly relevant to immune capacity. In detail, CD4 is the official designation for T-cell surface antigen T4/leu3. The functions of CD4 are to initiate or augment the early phase of T-cell activation. CD4 binds to relatively invariant sites on class II major histocompatibility complex (MHC) molecules outside the peptide-binding groove, which interacts with the T-cell receptor (TCR) [7,8]. Through its portion that resides inside the T cell, CD4 amplifies the signal raised from TCR by recruiting the lymphocyte-specific protein tyrosine kinase (LCK), which is essential for activating many molecules involved in the signaling cascade of an activated T cell. Buttini *et al*. [9] concluded that human CD4 may function as an important mediator of indirect neuronal damage in infectious and immune-mediated diseases of the central nervous system. The expression of human CD4 in microglia/macrophages creates a pathogenetic link between the immune system and the central nervous system. Based on their immunohistochemical features, CD4+ T cells are responsible for activating and directing other immune cells. They are essential in determining B cell antibody class switching, activating cytotoxic T cells, and maximizing bactericidal activity of phagocytes such as macrophages. Shedlock and Shen [10] also showed that CD4+ T cells are required in the priming phase for functional CD8 memory.

CD8 antigen is a cell surface glycoprotein found in most cytotoxic T lymphocytes that mediates efficient cell-cell interactions within the immune system. CD8 antigen, acting as a co-receptor, together with other TCRs on T lymphocytes, recognizes antigen processed by antigen presenting cells (APCs) in the context of class I MHC molecules [11]. The affinity between CD8 and the MHC molecule keeps the CD8+ T cells and the target cell bound tightly during antigen-specific activation. CD8+ T cells are capable of inducing the death of infected somatic or tumor cells; they kill cells which are infected with viruses (or other pathogens), or are otherwise damaged or dysfunctional.

In addition to the individual functions of CD4 and CD8, different combinations of them, *i.e*., CD4+CD8+, CD4+CD8-, CD4-CD8+ and CD4-CD8-, as well as the ratio of CD4+ to CD8+ also vary with health and disease status, and thus are highly relevant to immune capability of individuals. Specifically, CD4-CD8+ is MHC class I restricted and mainly recognizes replicating viral antigens, while CD4+CD8- is MHC class II restricted and responds to nonreplicating protein antigens processed by APCs [12-15]. The ratio of CD4+:CD8+ has been shown to be indicative of the general state of immune functioning, *e.g*., a high CD4+:CD8+ ratio may be indicative of improved immune activity [16,17]. In adult rats, differences in CD4:CD8 T cell ratios are MHC haplotype-dependent [18]. In human, Salazar *et al*. [19] reported that the CD4:CD8 ratio was a useful parameter in predicting HIV-TB co-infection. In swine, a specific feature is that a substantial number of both CD4-CD8- and CD4+CD8+ T cells were found in peripheral blood [15,20-24]. Summerfield *et al*. [25] demonstrated that CD4+CD8+ cells in swine can function as memory T-helper cells which proliferate upon stimulation with recall antigen.

QTL mapping has become a main tool for unraveling genetic mechanism of complex traits in livestock. Since the first study on QTL mapping in swine [26], more than 5,738 QTL have been identified for more than 558 traits http://www.animalgenome.org/QTLdb/pig.html. However, most of these QTL are relevant to production traits, and merely a small part of them [27-34] are relevant to immune-related traits. Although T lymphocyte subpopulations in peripheral blood have been recognized as a category of important immune traits, little attention has been paid to genetic basis of them, and solely a single study [27] was involved in QTL mapping for T lymphocyte subpopulations in peripheral blood in swine so far. Motivated by identifying the genetic control on T lymphocyte subpopulations and further assisting in selection for immune capacity in swine breeding, we performed herein a genome-wide scan for potential QTL influencing T lymphocyte subpopulations in peripheral blood in an experimental population involving three swine breeds.

## Results

### Alterations of proportions of T lymphocyte subpopulations in peripheral blood after challenge

The means and standard deviations of the proportions of T lymphocyte subpopulations or their ratios in peripheral blood on day 20 (the day before vaccinating) and day 35 (the day two weeks after vaccinating) are shown in Table 1. Compared with the measurements on day 20, the proportions of CD4+CD8-, CD4+ and the ratio of CD4+:CD8+ T cells in blood on day 35 reduced significantly, and the proportions of CD4-CD8+, CD4+CD8+ and CD8+ T cells in blood on day 35 increased significantly, but the proportion of CD4-CD8- T cells on day 35 changed only slightly and tended to retain after challenge.

**Table 1:** Means and standard deviations of the subpopulations of peripheral blood lymphocyte traits measured before (20-day-age) and 14 days after (35-day-age) challenge with live CSF (classical swine fever) vaccine

Trait	20-day-age	35-day-age	**P-value**^**a**^
Proportion of CD4+CD8+ T cells (%)	8.44 ± 3.94	10.62 ± 5.18	< 0.0001
Proportion of CD4+CD8- T cells (%)	19.12 ± 8.05	13.04 ± 7.71	< 0.0001
Proportion of CD4-CD8+ T cells (%)	37.27 ± 14.65	40.80 ± 12.10	0.0009
Proportion of CD4-CD8- T cells (%)	36.96 ± 34.03	34.85 ± 12.12	0.2960
Proportion of CD4+ T cells (%)	27.51 ± 9.05	24.53 ± 9.13	< 0.0001
Proportion of CD8+ T cells (%)	45.74 ± 15.14	51.37 ± 13.35	< 0.0001
Ratio of CD4+:CD8+ T cells	0.70 ± 0.39	0.52 ± 0.29	< 0.0001

^a^: Derived from t-test for comparing the differences between two ages

### QTL affecting T lymphocyte subpopulations in peripheral blood

**Figure 1:**
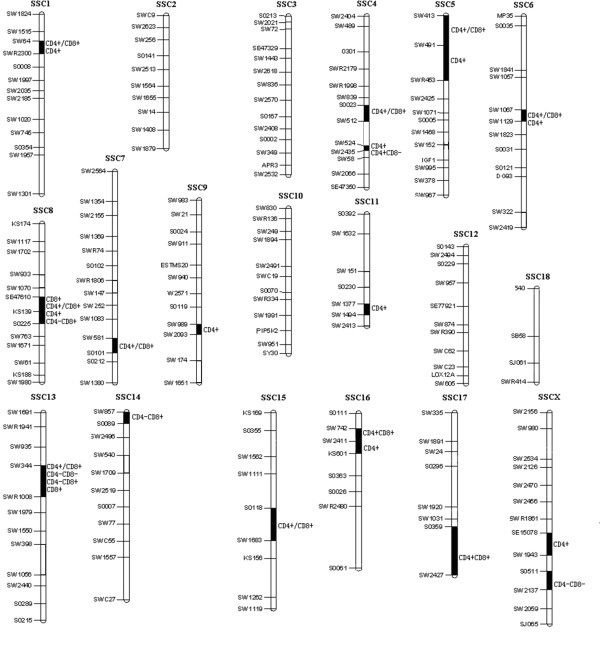
**Distribution of identified QTL among the genome**. This figure illustrates distributions of 27 detected QTL (FDR < 0.1) in the porcine genome for T lymphocyte subpopulations in peripheral blood.

The results of QTL analyses are compiled in Table 2. The distribution of identified QTL across the whole genome is also illustrated in Figure 1. A total of 27 QTL, including nine for proportion of CD4+ T cells, one for proportion of CD4+CD8- T cells, two for proportion of CD4+CD8+ T cells, two for proportion of CD4-CD8- T cells, three for proportion of CD4-CD8+ T cells, two for proportion of CD8+ T cells, and eight for ratio of CD4+: CD8+ T cells, were identified at the significance level of with FDR = 0.1. Some QTL on Sus Scrofa (SSC) Chromosomes 1, 4, 6, 8, 13 and 16 influenced more than one trait. On SSC8, four QTL for proportions of CD8+, CD4-CD8+, CD4+, and ratio of CD4+:CD8+ T cells, respectively, are located around marker KS139. On SSC13, the SW344-SW1008 region was found to harbor QTL for the proportions of CD4-CD8-, CD4-CD8+, CD8+ and ratio of CD4+:CD8+ T cells.

**Table 2:** Results of QTL mapping for the subpopulations of peripheral blood lymphocyte traits

Chromosome	Position^a^ (cM)	Trait	LR-value^b^	P-value^c^	FDR level^d^	Flanking	markers
						
						Left	Right
	24	CD4+	8.12	0.014	$	SW64	SWR2300
SSC1	24	CD4+	8.12	0.014	$	SW64	SWR2300
		CD4+/CD8+	13.46	0.001	**	SW64	SWR2300
SSC4	73	CD4+/CD8+	21.67	< 0.001	**	S0023	SW512
	100	CD4+CD8-	24.34	0.005	*	SW524	SW2435
		CD4+	30.18	< 0.001	**	SW524	SW2435
SSC5	1	CD4+/CD8+	10.5	0.006	$	SW413	SW491
	24	CD4+	8.62	0.009	$	SW491	SWR453
SSC6	76	CD4+	13.44	0.002	*	SW1067	SW1129
		CD4+/CD8+	10.08	0.008	$	SW1067	SW1129
SSC7	124	CD4+/CD8+	11.24	0.004	*	SW581	S0101
SSC8	65	CD4+	10.99	0.004	*	SE47610	KS139
		CD4+/CD8+	9.82	0.001	**	SE47610	KS139
	66	CD4-CD8+	7.46	0.02	$	KS139	S0225
		CD8+	10.37	0.003	*	KS139	S0225
SSC9	99	CD4+	6.93	0.018	$	SW989	SW2093
SSC11	73	CD4+	13.13	0.002	*	SW1377	SW1494
SSC13	30	CD4-CD8+	11.59	0.002	*	SW344	SWR1008
		CD4-CD8-	9.63	0.007	$	SW344	SWR1008
		CD8+	10.05	0.008	$	SW344	SWR1008
		CD4+/CD8+	7.6	0.016	$	SW344	SWR1008
SSC14	6	CD4-CD8+	7.79	0.02	$	SW857	S0089
SSC15	57.5	CD4+/CD8+	14.7	0.001	**	S0118	SW1683
SSC16	16	CD4+CD8+	9.83	0.007	$	SW742	SW2411
	17	CD4+	9.07	0.015	$	SW2411	KS601
SSC17	95	CD4+CD8+	8.41	0.014	$	S0359	SW2427
SSCX	84	CD4+	8.67	0.005	$	SE15078	SW1943
	104.8	CD4-CD8-	9.35	0.007	$	S0511	SW2137

^a^: Position corresponding to the peak of the LR profile

^b^: LR value at the peak of the LR profile

^c^: Derived from the empirical distribution obtained via 1,000 permutations.

^d^: $, FDR < 0.10; *, FDR < 0.05; **, FDR < 0.01.

## Discussion

Direct selection based upon observation of resistance and susceptibility to individual infections and diseases is generally infeasible [35]. The reason lies in two aspects: On the one hand, if the observation is made under normal production conditions, the phenotypes would not be very informative, because it is questionable to observe disease status of individuals with low disease resistance ability under good hygiene and management environment. On the other hand, if the observation is made under challenging conditions, it would be costly and productivity could be adversely influenced. T cells are the major cell populations mediating the adaptive arm of the immune system. Several studies on T cell subpopulations showed that variations in CD4 and CD8 T cell levels and CD4:CD8 ratio are significantly heritable [36-38]. Heritability estimates were around 65% for CD4:CD8 ratio, 50% for CD4+ counts, and 56% for CD8+ counts [38]. Ahmadi *et al*. [39] reported that heritability estimates are 54% for CD4+ counts, and 56%-62% for CD8+ counts. Therefore, as a category of immune-related traits with high heritabilities, T lymphocyte subpopulations can be possibly implemented to select for disease resistance and susceptibility in swine breeding. The present results clearly show that a number of loci contribute to the variation of T lymphocyte subpopulations in peripheral blood in pig. These findings would enhance our understanding of genetic control of the variations of T lymphocyte subpopulations.

In swine, very limited results on QTL mapping for T lymphocyte subpopulations in peripheral blood were reported so far. In the study of Wattrang *et al*. [27], a QTL for number of CD8+ cells and a QTL for number of CD2+ cells in a European Wild boar × Swedish Yorkshire inter-cross population were detected on SSC1. Within these QTL regions, no QTL was identified in our study, rather, two novel QTL, one for proportion of CD4+ T cells and the other for CD4:CD8 ratio, were mapped between SW64-SWR2300 on SSC1. This may be due to the difference of animal resources utilized in the two studies.

Compared with previous results of QTL mapping for immune related traits in swine, some QTL regions identified in this study are overlapping with those for other immune traits. Specifically, we identified two QTL for ratio of CD4+:CD8+ on SSC4 and SSC6, respectively. In the same chromosomal regions, two QTL for PWM-induced leukocytes proliferation were identified by Edfors-Lilja *et al*. [29]. Similarly, Reiner *et al*. [33] found a QTL on SSC13 for share of neutrophils and a QTL on SSC16 for share of basophils in pigs after challenge with Sarcocystis miescheriana. Overlapping with these two regions, four QTL with effects on proportions of CD4-CD8-, CD4-CD8+, CD8+ and CD4:CD8 ratio on SSC13, and two QTL on SSC16 for proportions of CD4+CD8+ and CD4+ T cells were determined in our study. These findings indicated that some immune-related traits in swine may be controlled by genes in tight linkage or same genes with pleiotropic effects. In addition, in our recent studies [28,34], a total of 46 QTL for two types of immune-related traits have been detected based on the same experimental population as in current study, including 11 QTL for three cytokine traits and 35 QTL for 18 haematological traits. These QTL are located across the whole genome except SSC18. Comparison of these QTL regions with those revealed in this study demonstrates that some of QTL underlying different immune-related traits trend to cluster within the same chromosomal regions. For example, QTL affecting level of IL-10, mean corpuscular hemoglobin (MCH), mean corpuscular hemoglobin concentration (MCHC) and red blood cell volume distribution width (RDW) were mapped within the interval of SW174-SW1651 on SSC9; Within two conjunct intervals of SW742-SW2411 and SW2411-KS601, QTL controlling MCHC, ratio of IFN-γ:IL-10 and proportions of CD4+CD8+, CD4+ were identified. This may suggest a common underlying mechanism predisposing the variation of certain immune-related traits.

In our earlier study [40], differences on T lymphocyte subpopulations among the three swine breeds have been investigated based on the same experimental animals, and the percentages of three types of T lymphocyte subpopulations, including CD4+CD8+, CD4+CD8-, and CD4-CD8-, were detected to be significantly different among the three pig breeds. This is the reason why we considered breed as a fixed factor in the statistical model to avoid potential confounding between effects of QTL and breed in present study. Hence, the statistical model adopted herein can suit well for the experimental design in this study because: 1) several previous studies [41-43] have demonstrated the advantages of a joint analysis across multiple populations with different genetic background over a single population analysis in QTL mapping; 2) all the animals of the three breeds involved in our study were reared on the same farm under the same conditions, ensuring no confounding or interaction between breed and environmental effects; and 3) the sample size of each breed is relatively small, and thus individual analyses within each breed are not feasible to obtain convincing results.

Some QTL reported in this study were mapped in the regions harboring several known genes, which have been reported in human or mice to have direct or indirect relationship with the traits considered in this research. Specifically, the QTL region on SSC1 for CD4:CD8 ratio harbors two genes, *TNFAIP3 *(*tumor necrosis factor, alpha-induced protein 3*) and *IFNGR1 *(*interferon gamma receptor 1*). *TNFAIP3 *in dendritic cells plays crucial role in controlling the magnitude of CD8+ and CD4+ T cell responses during both the priming and effector phases of immune response [44]. Tewari *et al*. [45] reported that the IFN-gamma receptor deficiency altered the epitope hierarchy of the pool of lymphocytic choriomeningitis virus-specific memory CD8 T cells in an acute infection. Another CD4:CD8 ratio QTL region on SSC4 harbors three genes, *i.e*., *IL6R *(*interleukin 6 receptor*), *CD1D *(*CD1d molecule*), and *TOX *(*thymocyte selection-associated high mobility group box*). *IL6R *is expressed on resting CD4+ and CD8+ cells, and its expression can significantly be enhanced on both activated CD4+ and CD8+ cells [46]. Thedrez *et al*. [47] showed that CD4 potentiates human iNKT cell activation by engaging CD1d molecules. Wilinson *et al*. [48] reported that transgenic mice that express TOX show expanded CD8+ and reduced CD4+ single positive thymocyte subpopulations. Two QTL for proportion of CD4+ and CD4:CD8 ratio, respectively, are located in the same region on SSC6. The *IL29 *(*interleukin 29*) gene is located in this region, which is an inhibitor of Th2 cytokine responses [49]. There are two genes, *IL2 *(*interleukin 2*) and *IL21 *(*interleukin 21*), which are located close to the QTL for proportion of CD4-CD8+ on SSC8. *IL2 *acts as a growth hormone for both B and T lymphocytes [50]. *IL21 *plays a global role in regulating T cell homeostasis [51]. On SSC15, a QTL for CD4:CD8 ratio was mapped to the region proximal to the *TLR3 *(*toll-like receptor 3*) gene. Salem *et al*. [52] suggested that CD8+ T cells can be activated by triggering their TLR3. The *ADA *(*adenosine deaminase*) gene is located close to the QTL for proportion of CD4+CD8+ on SSC17. Apasov *et al*. [53] found that the Ada -/- mice had a pronounced decrease in the size and lymphocyte content compared to the wild type mice.

It is well known that swine genome shows high homology to that of human and mice. From the viewpoint of comparative genomics, the same gene likely shows the similar function in swine, human and mice, which suggests that the genes aforementioned may serve as candidate genes for the traits of T lymphocyte subpopulations in swine. It is notable that QTL mapping performed in this study is the first step toward identification of the actual functional genes. Further endeavors focusing on the association between these genes and T lymphocyte traits will be pursued in our follow-up study.

## Conclusions

In swine, very few papers about QTL for T lymphocyte subpopulations in peripheral blood were available. In this study, 27 QTL with significance level of *FDR *< 0.10 were identified for 7 traits: two for CD4+CD8+, one for CD4+CD8-, three for CD4-CD8+, two for CD4-CD8-, nine for CD4+, two for CD8+, and eight for CD4+:CD8+ ratio. Within these QTL regions, a number of known genes were further revealed. Our results should be helpful for identifying the causal genes underlying these trait variations in swine.

## Methods

### The animals and collection of blood samples

The animals consisted of 367 piglets distributed in 5 Landrace boar families (15 sows and 87 piglets), 7 Large White boar families (33 sows and 190 piglets), and 4 Songliao Black Pig boar families (15 sows and 90 piglets), respectively. All pigs were raised under standard indoor conditions at the experimental farm of the Institute of Animal Sciences, Chinese Academy of Agricultural Sciences, Beijing, China.

All piglets were vaccinated with live CSF vaccine at 21 days of age. Blood samples were collected from each piglet one day before the vaccination inoculation (day 20) and two weeks after the vaccination (day 35), respectively. The samples were directly injected into eppendorf tubes containing 60 μl of 20% EDTA in phosphate-buffered saline (PBS).

### Identification of T lymphocyte subpopulations in peripheral blood

CD4+ and CD8+ T lymphocytes were obtained by the double cytofluorometric analysis. The blood cells were incubated with 10 μl of mouse anti porcine CD4-FITC (Serotec UK) and 10 μl of mouse anti porcine CD8-RPE (Serotec, UK) for 30 min, and then washed with 0.1 M PBS (pH 7.2, containing 0.3% bovine serum albumin). The red blood cells were digested with 0.1% ammonium oxalate solution. The stained cells were analyzed by cytofluorometry (Epicselite, Beckman-Coμlter, USA) to determine the CD4/CD8-defined T lymphocyte subpopulations: proportions of CD4+, CD8+, CD4+CD8+, CD4+CD8-, CD4-CD8+ and CD4-CD8-, and ratio of CD4+:CD8+.

### Genetic markers

206 microsatellites were selected from NCBI http://www.ncbi.nlm.nih.gov/ and the latest porcine sex-average linkage map in NCBI was used in QTL mapping. These markers are approximately evenly distributed throughout the 18 autosomes and the × chromosome. The average distance between adjacent microsatellites on the sex-averaged map was 12 cM. The number of markers and their mean polymorphic information content (PIC) [54] on each chromosome are shown in Table 3.

**Table 3:** Number of markers and their mean polymorphic information content (PIC)^a ^on each chromosome

Chr.	1	2	3	4	5	6	7	8	9	10	11	12	13	14	15	16	17	18	X
Number of markers	13	9	14	13	12	12	14	12	12	12	7	11	12	11	9	8	8	4	13
Mean PIC	0.63	0.68	0.61	0.54	0.49	0.65	0.63	0.57	0.51	0.53	0.58	0.47	0.55	0.62	0.56	0.55	0.55	0.45	0.51

^a^: The PIC for each marker was calculated as

$PIC=1-\overset{n}{\underset{i=1}{\sum }}P_{i}^{2}-\overset{n-1}{\underset{i=1}{\sum }}\overset{n}{\underset{j=i+1}{\sum }}2P_{i}^{2}P_{j}^{2}$

where *P_i _*is the frequency of the ith allele, *P_j _*is the frequency of the jth allele and n is the number of animals in the population.

### Statistical analysis

Interval mapping of QTL was performed using the variance component approach [55-57] based on a linear mixed model as follows,

y=Xa+bc+Zu+Tv+e

$$u\sim \text{N}\left(0,\;A\sigma_{u}^{2}\right),\;v\sim \text{N}\left(0,\;Q\sigma_{v}^{2}\right),\;e\sim \text{N}\left(0,\;I\sigma_{e}^{2}\right),\;Cov\left(u,\;v^{\prime }\right)=0$$

where **y **is a vector of the phenotypic values for the proportions of peripheral blood lymphocyte or their ratios measured on day 35, **a **is a vector of fixed effects including breed, sex and sampling batch, **c **is a vector of the proportions of peripheral blood lymphocyte or their ratios measured on day 20, *b *is the regression coefficient, **u **is a vector of residual polygenic effects, **v **is a vector of QTL allelic effects, **e **is a vector of residuals, **X**, **Z**, and **T **are incidence matrices for **a**, **u**, and **v**, respectively, **A **is the additive genetic relationship matrix among all individuals, $\sigma_{u}^{2}$ is the additive polygenetic variance, **Q **is the IBD probability matrix among QTL alleles which was constructed based on multi-locus marker genotypes using the Monte-Carlo approximation method proposed by Grignola *et al*.[57], $\sigma_{v}^{2}$ is the QTL allelic variance, **I **is an unit matrix, and $\sigma_{e}^{2}$ is the residual variance.

The QTL analysis was scanned along each chromosome at every interval of 1 cM. Restricted maximum likelihood (REML) was used to estimate the three variance components in the model and the likelihood ratio (LR) was calculated as test statistic for each particular location on the chromosome as follows

$$LR=-2\ln\frac{L_{\text{MAX}}|{\text{H}}_{0}}{L_{\text{MAX}}|{\text{H}}_{\text{A}}}$$

where *L*_MAX_|H_0 _and *L*_MAX_|H_A _are restricted maximum likelihood functions corresponding to the null hypothesis (there is no QTL) and the alternative hypothesis (there is a QTL), respectively.

The program MQREML developed by Zhang and Hoeschele [56] was used for the calculation mentioned above.

When the distribution of the phenotypes is normal or nearly normal, it is generally regarded that the LR statistic follows approximately a χ^2 ^distribution with degrees of freedom between one and two. However, the distributions of the traits measured in this study are severe departing from normal, and a data transformation using Box-Cox approach does not help much. So, we adopted the permutation approach [58] to obtain the empirical distribution of the LR statistic. For each chromosome and each trait, 1,000 permutations of the phenotypes were performed and from the empirical distributions of the LR-values the probability corresponding to the observed LR value was obtained. In order to avoid the increase of false positive rate caused by multiple tests, the FDR (false discovery rate) control approach [59] was adopted to determine the significance levels for declaring significant QTL. Compared with the traditional method of controlling family wise error rate (FWER), such as Bonferroni correction, the FDR method is less conservative, leading to an essential gain in power. Thus FDR is increasingly being adopted in area of QTL mapping experiments.

Let *m *be the total number of tests involved in the analysis and *P*_1 _≤ *P*_2 _≤···≤ *P*_m _be the ordered observed *P*-values corresponding to the empirical distributions for the *m *tests, the *FDR *for *P_i _*(*i *= 1, 2,..., *m*) is calculated as

$$FDR=\frac{mP_{i}}{i}$$

In this study, there were 7 phenotypic traits and 19 chromosomes, leading to the total number of tests *m *= 7 ×19 = 133.

## Competing interests

The authors declare that they have no competing interests.

## Authors' contributions

XL and JFL are the major executive persons of all jobs of this research and drafted this manuscript together. YFG assisted in the collection of the phenotypes and marker genotyping. ZPW assisted in the statistical analysis. YL assisted in phenotype collecting. QZ planed and supervised the whole study. All authors read and approved the final manuscript.
